# Supplementary material for: Role and mechanism of NCAPD3 in promoting malignant behaviors in gastric cancer
Source: Front Pharmacol. 2024 Apr 22;15:1341039. doi: 10.3389/fphar.2024.1341039 (PMC11070777; doi:10.3389/fphar.2024.1341039)
Supplement: Supplementary file 11 [file DataSheet2.ZIP › GSEA/immunologic/my_analysis.Gsea.1599462003817/GSE21360_PRIMARY_VS_TERTIARY_MEMORY_CD8_TCELL_DN.html]

Details for gene set GSE21360\_PRIMARY\_VS\_TERTIARY\_MEMORY\_CD8\_TCELL\_DN[GSEA]

|  || Dataset | filtered\_dataset.sample\_info.cls#WT\_versus\_NCAPD3\_MUT |
| Phenotype | sample\_info.cls#WT\_versus\_NCAPD3\_MUT |
| Upregulated in class | NCAPD3\_MUT |
| GeneSet | GSE21360\_PRIMARY\_VS\_TERTIARY\_MEMORY\_CD8\_TCELL\_DN |
| Enrichment Score (ES) | -0.49674955 |
| Normalized Enrichment Score (NES) | -2.6393807 |
| Nominal p-value | 0.0 |
| FDR q-value | 0.0035396046 |
| FWER p-Value | 0.018 |
Table: GSEA Results Summary

  

Fig 1: Enrichment plot: GSE21360\_PRIMARY\_VS\_TERTIARY\_MEMORY\_CD8\_TCELL\_DN      
 Profile of the Running ES Score & Positions of GeneSet Members on the Rank Ordered List

  

| SYMBOL | TITLE | RANK IN GENE LIST | RANK METRIC SCORE | RUNNING ES | CORE ENRICHMENT || 1 | 1497 | CTNS | 58 | 0.883 | 0.0117 | No |
| 2 | 8454 | CUL1 | 492 | 0.477 | -0.2729 | No |
| 3 | 2140 | EYA3 | 528 | 0.458 | -0.2704 | No |
| 4 | 23294 | ANKS1A | 768 | 0.339 | -0.4229 | No |
| 5 | 6385 | SDC4 | 871 | -0.282 | -0.4796 | Yes |
| 6 | 5292 | PIM1 | 885 | -0.300 | -0.4708 | Yes |
| 7 | 7453 | WARS | 900 | -0.325 | -0.4612 | Yes |
| 8 | 4616 | GADD45B | 904 | -0.330 | -0.4433 | Yes |
| 9 | 2037 | EPB41L2 | 943 | -0.365 | -0.4486 | Yes |
| 10 | 3665 | IRF7 | 956 | -0.375 | -0.4345 | Yes |
| 11 | 4864 | NPC1 | 964 | -0.384 | -0.4163 | Yes |
| 12 | 2146 | EZH2 | 1001 | -0.406 | -0.4177 | Yes |
| 13 | 9308 | CD83 | 1025 | -0.423 | -0.4086 | Yes |
| 14 | 10616 | RBCK1 | 1042 | -0.438 | -0.3936 | Yes |
| 15 | 7132 | TNFRSF1A | 1055 | -0.449 | -0.3750 | Yes |
| 16 | 1543 | CYP1A1 | 1058 | -0.451 | -0.3491 | Yes |
| 17 | 3669 | ISG20 | 1097 | -0.475 | -0.3478 | Yes |
| 18 | 3556 | IL1RAP | 1099 | -0.476 | -0.3196 | Yes |
| 19 | 6947 | TCN1 | 1135 | -0.503 | -0.3144 | Yes |
| 20 | 27074 | LAMP3 | 1158 | -0.522 | -0.2986 | Yes |
| 21 | 2152 | F3 | 1188 | -0.559 | -0.2856 | Yes |
| 22 | 10221 | TRIB1 | 1229 | -0.604 | -0.2779 | Yes |
| 23 | 837 | CASP4 | 1245 | -0.623 | -0.2509 | Yes |
| 24 | 8651 | SOCS1 | 1272 | -0.661 | -0.2295 | Yes |
| 25 | 8744 | TNFSF9 | 1305 | -0.708 | -0.2097 | Yes |
| 26 | 253782 | CERS6 | 1307 | -0.711 | -0.1673 | Yes |
| 27 | 7706 | TRIM25 | 1357 | -0.817 | -0.1531 | Yes |
| 28 | 6711 | SPTBN1 | 1381 | -0.925 | -0.1136 | Yes |
| 29 | 5055 | SERPINB2 | 1397 | -1.088 | -0.0583 | Yes |
| 30 | 8548 | BLZF1 | 1398 | -1.103 | 0.0087 | Yes |
Table: GSEA details [plain text format]

  

Fig 2: GSE21360\_PRIMARY\_VS\_TERTIARY\_MEMORY\_CD8\_TCELL\_DN      
 Blue-Pink O' Gram in the Space of the Analyzed GeneSet

  

Fig 3: GSE21360\_PRIMARY\_VS\_TERTIARY\_MEMORY\_CD8\_TCELL\_DN: Random ES distribution      
 Gene set null distribution of ES for **GSE21360\_PRIMARY\_VS\_TERTIARY\_MEMORY\_CD8\_TCELL\_DN**
